# Finite Element Modeling for Virtual Design to Miniaturize Medical Implants Manufactured of Nanostructured Titanium with Enhanced Mechanical Performance

**DOI:** 10.3390/ma15217417

**Published:** 2022-10-22

**Authors:** Nikita Kazarinov, Andrey Stotskiy, Alexander Polyakov, Ruslan Z. Valiev, Nariman Enikeev

**Affiliations:** 1 Institute of Problems of Mechanical Engineering, 199178 St. Petersburg, Russia; 2 Dynamics and Extreme Characteristics of Promising Nanostructured Materials, Saint Petersburg State University, 199034 St. Petersburg, Russia; 3 Institute of Physics of Advanced Materials, Ufa State Aviation Technical University, K. Marx 12, 450008 Ufa, Russia; 4 Laboratory of Multifunctional Materials, Bashkir State University, 450076 Ufa, Russia; 5 Center for Design of Functional Materials, Bashkir State University, 450076 Ufa, Russia

**Keywords:** ultrafine-grained materials, medical implants, fatigue, mechanical properties, titanium, finite element modeling

## Abstract

The study is aimed to virtually miniaturize medical implants produced of the biocompatible Ti with improved mechanical performance. The results on the simulation-driven design of medical implants fabricated of nanostructured commercially pure Ti with significantly enhanced mechanical properties are presented. The microstructure of initially coarse-grained Ti has been refined to ultrafine grain size by severe plastic deformation. The ultrafine-grained (UFG) Ti exhibits remarkably high static and cyclic strength, allowing to design new dental and surgical implants with miniaturized geometry. The possibilities to reduce the implant dimensions via virtual fatigue tests for the digital twins of two particular medical devices (a dental implant and a maxillofacial surgery plate) are explored with the help of finite element modeling. Additionally, the effect of variation in loading direction and the fixation methods for the tested implants are studied in order to investigate the sensitivity of the fatigue test results to the testing conditions. It is shown that the UFG materials are promising for the design of a new generation of medical products.

## 1. Introduction

The Commercially Pure Titanium (CP Ti) and Ti-based alloys belong to the class of biomaterials which are massively used in medical applications in dental [1] and maxillofacial surgery [2] due to their remarkable advantages. Ti materials are characterized by the combination of outstanding corrosion resistance, osseointegration property and biocompatibility demanded for manufacturing long-term implants [3,4].

Fatigue failure represents a major problem for medical implants from a mechanical point of view; the screw thread section is the most troublesome zone for dental implants [4,5]. To prevent undesired early failure, the implants should exhibit high mechanical strength to sustain considerable static and cyclic physiological loading. On the other hand, there are natural geometric and size limitations implied by human anthropometry, and many specific surgical treatments require application of implants with reduced dimensions [6]. Therefore, the studies aimed at miniaturization of the surgical appliances are of the great demand [6,7].

Among the medical Ti-based alloys, CP Ti contains the least concentration of impurities, while relatively low elastic modulus of this material reduces stress shielding, and hence, improves the implant-bone bonding. However, relatively low static strength and fatigue properties of the CP Ti limit its applicability in medical products. The mechanical performance of the pure Ti can be notably improved via alloying, as is done for the popular Ti6Al4V alloy. However, vanadium or aluminum additions might appear to be toxic [7], and despite attractive mechanical and comparable biological properties of the Ti alloys, further clinical studies comparing the long-term performance of the CP Ti, Ti6Al4V and other alloys are required [8].

An alternative approach to increase the mechanical performance of CP Ti is associated with the microstructural design of a selected material without variation in its chemical composition. Recently developed techniques of severe plastic deformation (SPD) are capable of imposing extremely high strains into processed metallic workpieces, leading to significant grain refinement and fabrication of ultrafine-grained (UFG) materials with nanostructural features, significantly enhancing multifunctional properties often defined as nanostrcutured materials [9,10]. SPD techniques such as Equal Channel Angular Pressing (ECAP) [11] allow producing bulk UFG workpieces of various metals and alloys. On that basis, specially developed SPD-based procedures lead to producing long-sized billets of nanostructured CP Ti [12] with significantly improved mechanical performance and biocompatibility, and thus, they are highly attractive for manufacturing advanced medical implants with reduced dimensions [13,14]. Grain refinement processes impose unique positive effects on cell adhesion providing better proliferation, osteogenic differentiation and mineralization of various types of cells [15].

However, effective engineering of new medical implants accounting for improved mechanical parameters of the UFG materials is hardly achievable without the application of reliable predictive computer-aided solutions. The growing progress in simulation technologies promotes their intensive usage for the design of various medical products to be composed of new materials. Finite element method (FEM) is proved to be a powerful technique for development of implants with optimized design, considering the variation of implant dimensions and connections [16], addressing various problems of oral and maxillofacial biomechanics [17], capturing the effect of thread shape and inclination [18], etc. Encouraging results have been recently achieved in experimentally justified FEM-driven study to develop narrow [19] and extra-narrow dental implants [20] and in numerical examination of the effect of ultrafine-grained structures on the fatigue limit for dental implants [21]. However, the efforts to account for the enhanced mechanical performance of nanostructured materials to miniaturize the implant dimensions have been rarely reported in the literature.

Note that the pioneer miniaturized dental implants with reduced diameter have already been engineered using the CP UFG Ti by the Timplant Ltd. Company (Ostrava, Czech Republic) [22] on the basis of a multi-disciplinary research and development of nanostructured titanium-based materials for medical applications [23]. This achievement allowed, for the first time, the implantation of a CP-Ti dental implant with reduced dimensions into the front teeth area of a patient at a one-stage surgery procedure avoiding more risky surgery, such as alveolar splitting or augmentation [13].

However, purposeful studies to identify the limits for the further miniaturization of the implants relying on the enhanced properties of the nanostructured material is rarely reported in the literature. The simulation-aided approaches for virtual testing of versatile configurations of new nano-Ti medical articles keeping their reliable long-term functioning seem to be very important to develop. Based on the previous experience [24], we present a FEM-based numerical study to feature the virtual design of medical implants manufactured of nanostructured Ti with enhanced mechanical properties. The novelty of this research is related to a combination of approaches considering the enhanced material and applying the technique for its virtual testing in the form of the unique components, which can hardly be fabricated of traditional CP Ti. As a result, we propose pathways to miniaturize the medical devices, keeping their biomechanical performance and reliable functioning at the industrially required level. We demonstrate the viability of our approach using virtual testing of a dental implant and a maxillofacial surgery plate with reduced dimensions as an example.

## 2. Materials and Methods

### 2.1. Experimental

**Figure 1:**
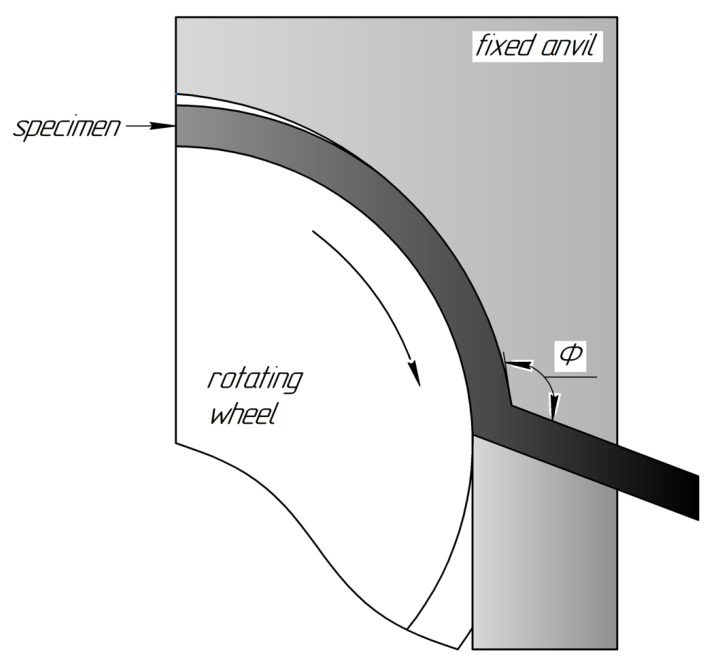
Schematic representation of the ECAP-C processing.

The CP Ti Grade 4 was chosen as the object of investigation. CP Ti of Grade 4 manufactured by Dynamet Company (Washington, PA, USA) was received as hot-pressed rods with a diameter of 12 mm. The chemical composition according to a certificate (confirmed also by the spectral analysis) was as follows: Ti-base, 0.04% C, 0.14% Fe, 0.006% N, 0.36% O, 0.0015% H. The UFG structure in the CP Ti was achieved by a multi-stage procedure involving SPD by continuous ECAP-Conform (ECAP-C) technique followed by wire drawing. The ECAP-C implements the idea of permanent feeding the ECAP with rods of unlimited length [25], demonstrating a promising potential for grain refinement in the long-sized workpieces [26]. A schematic view of the ECAP-C process is presented in Figure 1. This technology being combined with subsequent wire drawing proved to produce long-sized UFG billets with enhanced properties [12] and dimensions suitable for manufacturing of implants [13,14].

In order to achieve the UFG structure, we processed CG Ti rods with a diameter of 12 mm by the ECAP-C device at a temperature T = 200 °C. The number of passes was n = 6 and the angle of the channels’ intersection (see Figure 1) was Φ = 120°. The output workpieces were additionally subjected to drawing at the same temperature to manufacture long-sized UFG Ti rods with the diameter of 6 mm (see details in [13,14]).

TEM observations of microstructure were carried out using JEM 2100 microscope (JEOL Ltd., Tokyo, Japan). Thin foils for the TEM investigations were prepared by double-sided blast polishing using Tenupol 5 device (Struers LLC, Cleveland, OH, USA).

Tensile tests were performed with an INSTRON machine (ToolWorks Inc., Norwood, MA, USA) at a strain rate of 10^−^^3^ s^−1^. The mechanical properties were evaluated according to the STM E8-13a standard. At least three specimens per state were tested to ensure the reliability of the measurements.

**Figure 2:**
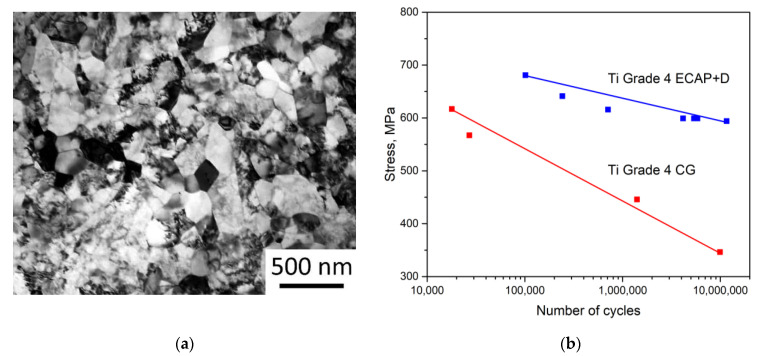
TEM image of the refined microstructure of the CP Ti Grade 4 produced by ECAP-C followed by drawing (**a**); S-N curves for the CG Grade 4 Ti and the UFG Grade 4 Ti reconstructed within ANSYS Workbench FEM software (Canonsburg, PA, USA) in accordance to the data presented in [13] (**b**).

The main goal of the experimental efforts was to achieve the input data on the mechanical parameters of nanostructured Ti to be used within the FEM simulation procedures and to ensure reproducibility and consistency with earlier results, related to application of nano-Ti in medical implants [13,14]. As shown by TEM observations, after ECAP-C followed by drawing treatment, the grain size (*d*) of the CP Ti was significantly reduced: from *d*~25 µm down to *d*~150 nm (Figure 2a). Such a drastic grain refinement provided a significant enhancing the mechanical properties, which is in a good agreement with earlier studies [13,14]. The ultimate tensile strength increased from 730 to 1255 MPa and the yield stress—from 500 to 1200 MPa for CG and nano-Ti, respectively. These parameters were used to represent the properties of CP Ti in different structural states during FEM simulations. The improved cyclic mechanical properties were accounted by the analysis of fatigue strength (S-N) curves achieved in [13] and incorporated into the FEM formalism, as presented in Figure 2b.

The parameters of mechanical behavior of Ti in the CG and the UFG states used in the present numerical study are summarized in Table 1. The material’s constants are represented by the reference values, while the strength parameters have been set according to the conducted tensile tests.

### 2.2. Numerical Simulation

We used FEM throughout the conducted study to design various medical implants, as denoted in the introduction. We built three-dimensional models to digitally test the implants using CAE software KOMPAS-3D (Ascon, Moscow, Russia) to be imported into the ANSYS Workbench FEM software suite (Canonsburg, PA, USA). During computations, the material was supposed to exhibit linear and elastic behavior with the parameters listed in Table 1. Virtual fatigue testing was carried out using the built-in fatigue module and the S-N curves presented in Figure 2b combined with a mean stress correction theory. In order to obtain mesh-independent results, the mesh sensitivity testing was performed for all the models: the finite element mesh was refined to the point when further reduction of the characteristic element size did not affect the computational results. As a result, all the dental implant models were meshed with tetrahedral four-node elements. The number of elements for the dental implant models ranged from 400,910 (1.8 mm model) to 728,819 (2.4 mm base model). Meshes for the maxillofacial surgery plate models was composed of brick elements; the element number ranged from 48,394 (model with the cross-section area reduced by 60%) to 73,030 (for the base model).

To assess possible ways to miniaturize the implants, the device with a standard geometry was supposed to be manufactured of the standard CG Ti Grade 4. The standard CG Ti model was loaded in a way to obtain nearly critical stress state both in terms of static and fatigue failure. Afterwards, this load was applied to models with reduced dimensions but composed of the UFG Ti. Details on boundary conditions and loading parameters for the case of each implant are given in Section 2.2.1 and Section 2.2.2.

#### 2.2.1. One-Stage Dental Implant

**Figure 3:**
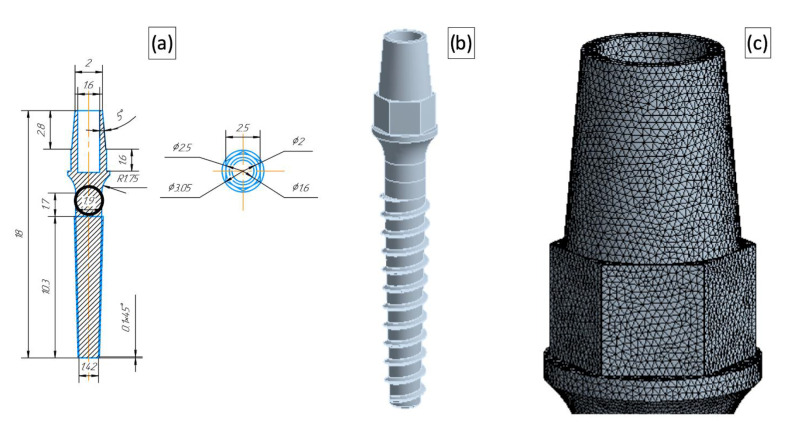
Dental implant model with a reduced diameter (1.9 mm). A technical drawing (**a**) with the diameters used for the model differentiation put into circles, where the dimensions are given in mm; The 3D CAD model (**b**); A magnified view of the FEM mesh (**c**).

A one-stage dental implant with a generic geometry was considered in the study. The configuration of the base model corresponded to the geometry designed by the Timplant Ltd. Company [22] for an implant with a diameter of 2.4 mm. In this numerical study, we varied the implant diameter in the range of 1.8–2.4 mm. A technical drawing of the miniaturized dental implant (with the diameter of 1.9 mm) with a corresponding CAD model and the detailed representation of the finite element mesh are presented in Figure 3.

**Figure 4:**
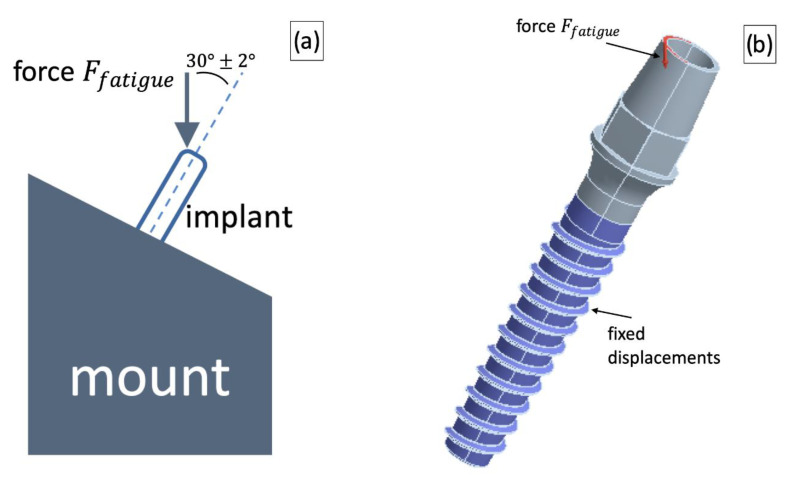
A loading scheme used for testing dental implants (**a**) and the corresponding CAD implementation (**b**).

The loading scheme for the dental implant was reproduced to meet the testing procedures used in the ISO 14801:2012 standard. The implant was fixed in a stationary foundation and loaded with a force applied at the angle of 30 ± 2° with respect to the implant axis. These boundary and loading conditions were simulated by imposing displacement restrictions in the implant thread area and force application to the upper edge of the implant at the angle of 30 ± 2°with respect to the device axis. The loading scheme and corresponding boundary conditions are shown in Figure 4.

Note that the available experimental fatigue data were obtained for a fully reversed load setup, while operation of the dental implant was supposed to correspond to a zero-based loading (and therefore, to a non-zero mean stress), since the chewing effect was simulated. This task definition required application of a mean-stress correction theory; accordingly, the Soderberg correction [27] was used which is often regarded as the most conservative approach [28]. The Soderberg mean stress correction theory implies the following relation between actual stress amplitude $\sigma_{a}$, the mean stress $\sigma_{m}$, the material yield stress $\sigma_{y}$ and the stress amplitude $\sigma_{w}$ used in the experiments with a fully reversed load to assess the S-N curve:

(1) $$\frac{\sigma_{m}}{\sigma_{y}}+\frac{\sigma_{a}}{\sigma_{w}}=1$$

Expression (1) can be used to relate actual loading parameters ($\sigma_{a}$ and $\sigma_{m}$) to the stress amplitude from the experiments $\sigma_{w}$ and in this way evaluate the limiting load cycle number using the S-N curves. The conducted virtual fatigue tests were based on accounting for the maximal principal stress.

The force value for the fatigue tests simulations $F_{fatigue}$ was chosen so that the CG Ti implant with the standard geometry could safely sustain the cyclic load—the model is able to sustain more than 2 × 10^6^ loading cycles (in accordance with the ISO 14801:2012 standard). Afterwards, the force $F_{fatigue}$ was applied to the UFG Ti implant models with reduced dimensions, and the number of cycles was evaluated for this load. The model was considered to be safe if it passed 2 × 10^6^ cycles. Additionally, sensitivity to the load application method was studied: the force application angle was varied to be consistent with the recommendations of the ISO 14801:2012 standard (loading application angle of 30 ± 2°), and therefore, virtual fatigue tests were conducted using three force application angles—28°, 30° and 32°.

The static strength virtual tests were conducted in a similar manner. Firstly, the critical force $F_{static}$ was evaluated for the CG Ti implant with the basic geometry: $F_{static}$ corresponded to the case when maximal principal stress $\sigma_{1}^{max}$ matched yield stress $\sigma_{y}$ of the CG Ti (530 MPa). Furthermore, the $F_{static}$ force was applied to the models with a reduced diameter and $\sigma_{1}^{max}$ was also examined. The model was considered to be safe if the $\sigma_{1}^{max}$ value did not exceed the yield stress of the UFG Ti (1200 MPa). In this case, the force application angle was not varied and the value of 30° was used.

#### 2.2.2. Maxillofacial Surgery Plate

A bone-shaped surgical plate produced by the company Conmet LLC (Moscow, Russia) [29] was chosen as a prototype of the base model. In this case, the model accounted for the symmetry of the device and only half of the plate was simulated by application of proper symmetry conditions. The standard model dimensions were varied in order to simulate devices with the reduced area of the cross-section. Models with the cross-section area reduced by 20% (model name UFG20), 30% (model name UFG30) and 40% (model name UFG40) were tested. The device thickness and width were reduced proportionally; however, the fixation holes were kept identical for all the models.

**Figure 5:**
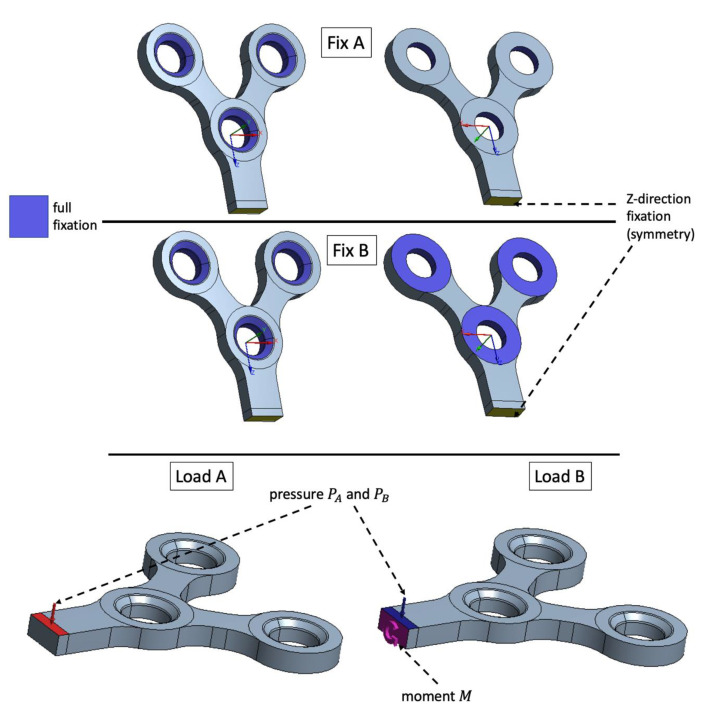
Investigated fixation and loading methods: Fix A—fixation with screws only; Fix B—fixation with screws and plate-bone adhesion in the vicinity of the mounting holes. Pressure applied to the plate’s top is denoted as Load A and pressure combined with twist loading with the moment *M* is denoted as Load B.

For the case of the maxillofacial plate, several types of boundary conditions and loads were investigated, since different variations in fixation methods can be successively used in craniofacial surgery depending on the clinical needs [30]. The standard testing procedures for the surgery plates suppose relatively simple three-point bending testing technique, which is able to provide only a basic insight into the surgical device strength. Using the numerical technique, it is possible to investigate more complicated loads and boundary conditions which could provide additional information on the device behavior in real operating conditions. It was supposed that in the early post operational stages the plate is fixed only by screws and is not attached to the bone (Fixation A in Figure 5). The second fixation type supposed adhesion between the device and the bone in the vicinity of the fixation holes and this way Fixation B (Figure 5) boundary conditions were applied.

Two types of loading were studied: pressure applied to the plate top (Load A in Figure 5) and pressure on the plate top combined with the moment applied to the cross section of the plate to simulate twist loading (Load B in Figure 5).

The load values $P_{A},$ $P_{B}$ and M were chosen in the same way as for the dental implant case: the device with a standard geometry and fixation A (Figure 5) should withstand 2 × 10^6^ load cycles. Afterwards, the evaluated loads were applied to the devices with the reduced dimensions. Additionally, for each load type, the fixation B was investigated using the same $P_{A},$ $P_{B}$ and M load values simulating adhesion of the plate to the bone.

## 3. Results

We applied the FEM-based approach to optimize the design of medical articles manufactured of a material with enhanced mechanical properties. The implants used for dental maxillofacial surgery were miniaturized and details of these two case studies are reported below.

### 3.1. Dental Implant

The $F_{fatigue}$ was evaluated using virtual fatigue testing of the CG Ti dental implant with standard dimensions. The assessed $F_{fatigue}$ was 75.6 N resulting into 2,075,000 load cycles before the device failure. This value was further used for the virtual fatigue testing of the dental implants with reduced dimensions.

**Figure 6:**
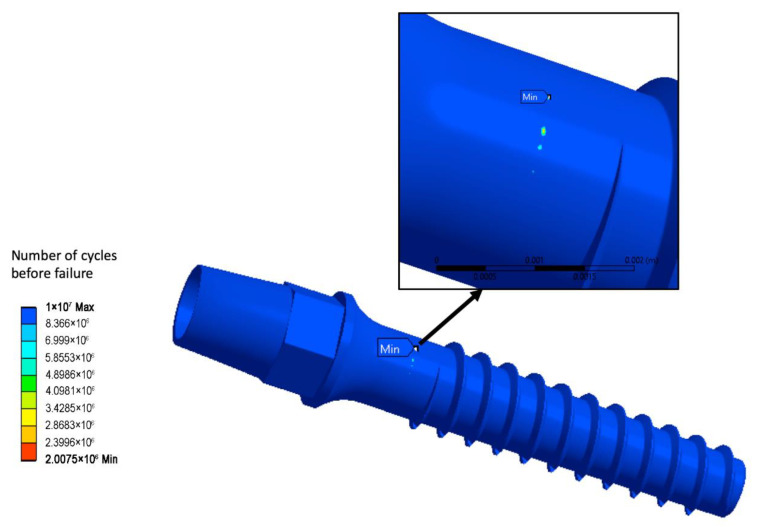
Life prediction for the CG Ti implant with a standard geometry.

Figure 6 shows numerical prediction of the fatigue life for the standard geometry CG Ti implant when $F_{fatigue}$ = 75.6 N is applied. The troublesome zone with the minimal life of 2,075,000 cycles, and hence, with the potential risk of fracture initiation after this number of cycles, is indicated.

The evaluated static load force $F_{static}$ was 84.64 N. In the standard geometry model, such a load corresponded to the maximal principal stress $\sigma_{1}^{max}$ of 530 MPa, and this value was further used in the simulation of static strength tests for devices with reduced dimensions.

**Figure 7:**
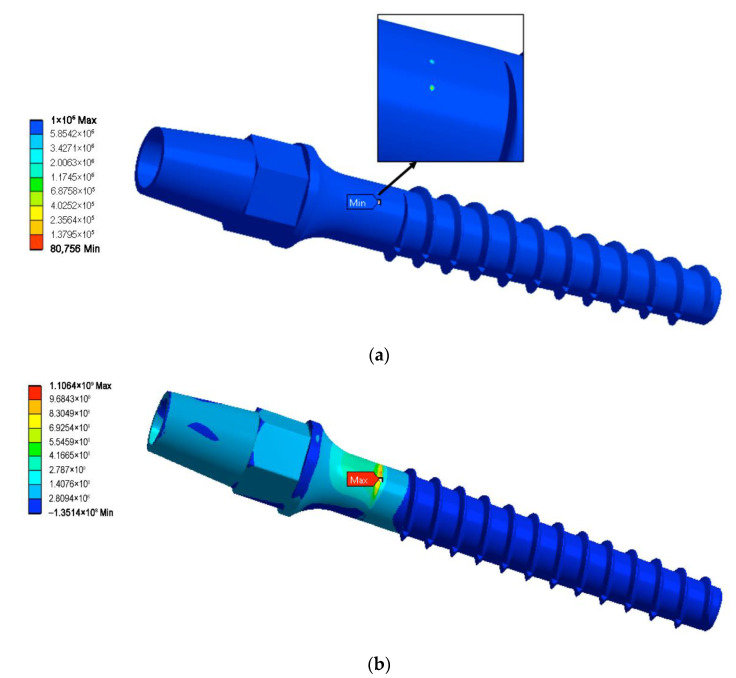
Life prediction for the 1.9 mm UFG Ti model and force application angle of 32° (**a**) and maximal principal stress $\sigma_{1}^{max}$ distribution in the static load test for the 1.8 mm model (**b**).

Table 2 contains fatigue strength computation results for all geometries of the dental implant and three load angles. The conducted computations reveal that even slight variation of the force application angle can potentially change the minimally admissible implant diameter. Application of the UFG Ti alloy allows safe diameter reducing down to two millimeters: the two-millimeter model sustained the maximal number of load cycles for all three force application angles. Further reduction of the device diameter (down to 1.9 mm) was shown to be troublesome, since the 1.9 mm UFG implant was able to withstand an acceptable number of the load cycles for two load angles—the basic one (30°) and 28°. However, increasing the force application angle up to 32° led to the model failure at only 80,756 load cycles, which is far below the 2 × 10^6^ limit. Thus, the 1.9 mm model can be considered as partially acceptable. The 1.8 mm model was not reliable enough to pass the virtual fatigue tests. Figure 7a shows the fatigue life prediction for the 1.9 mm model loaded with a 32° force application angle as an example of the fatigue computations results.

The static strength virtual tests reveal that all the miniaturized UFG Ti models sustained the $F_{static}$ load force (84.645 N, application angle of 30°), since for all of them, the maximal principal stress was below the yield stress of the UFG Ti. Table 3 presents values of the maximal static stress for all studied models, while Figure 7b shows the distribution of the maximal principal stress for the smallest 1.8 mm diameter model.

According to the conducted computations, the dental implant diameter can be safely reduced by about 16.6% from 2.4 mm to 2.0 mm if it is fabricated of the UFG Ti. Further reduction is theoretically possible; however, the fatigue test can be failed for certain force application angles which fit the considered ISO standard range (30 ± 2°).

### 3.2. Maxillofacial Surgery Plate

The following load values were assessed using the above-described load evaluation procedure: $P_{A}=27.72\;\mathrm{MPa},$ $P_{B}=1.53\;\mathrm{MPa}$ and M=0.0834 Nm. These loads resulted in 10^6^ load cycles before failure of the CG Ti plate with the standard dimensions and type A fixation method.

For the different plate geometries, two fixation methods and two load variants were investigated resulting to sixteen virtual fatigue tests. The maxillofacial surgery plate fatigue test results are listed in Table 4. All results were obtained for the same loading values, i.e., $P_{A}=27.72\;\mathrm{MPa},$ $P_{B}=1.53\;\mathrm{MPa}$ and M=0.0834 Nm.

**Figure 8:**
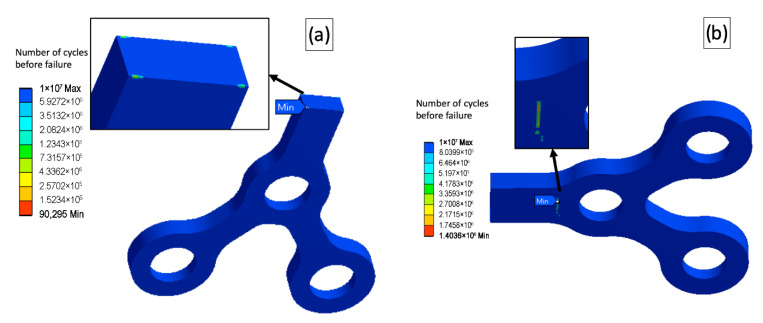
Location of zones where fatigue fracture can be possibly initiated; (**a**)—model UFG30, load B, fixation A; (**b**)—base model, load A, fixation B.

As is seen from the conducted tests, the type B fixation combined with the complex load B led to premature failure for all the models, including the base one. However, the UFG20 model clearly outperformed the base model in all other “load-fixation” combinations sustaining the maximal number of cycles before failure (10^7^), showing a reasonable potential for the additional reducing of the model’s dimensions. The further miniaturized plate (UFG30) passed a simple load test (load A), but failed complex load tests. Therefore, the affordable maximal cross-section reduction can be about 25%. The load and the fixation types influenced dramatically the fatigue behavior of the models and the locations of the potentially troublesome zones sustaining the low number of load cycles before fracture. Figure 8 shows several examples of the virtual fatigue tests and different locations of zones with low fatigue strength.

## 4. Discussion

The FEM-based modeling proved to be a powerful tool for design and engineering of various medical articles—from dental implants to hip joints [31,32]. The fatigue life predictions using FEM for the Ti implants were proven to show good consistence with the experimental validation results [20,21,33,34]. The formalism used in the present work is based on reliable numerical procedures for the static and cyclic loading conditions accounting for the improved mechanical parameters of the considered material. Since the nanostructured Ti demonstrates excellent mechanical performance as compared to its coarse-grained counterpart, the possibility for optimization of the product design is open. Enhanced static and cyclic strength of the nano-Ti combined with relatively low elastic modulus allow one to considerably reduce dimensions of the medical surgical appliances.

The conducted numerical computations show the limits for miniaturization of the medical implants produced of the enhanced biomaterials. Note that accounting for deviation in loading conditions may affect the fatigue numerical test. For example, even a slight alternation of the force application angle in the fatigue test of the dental implant may result in the undesired number of load cycles before failure. The minimal dental implant diameter for reliable long-term functioning was figured out accounting for these issues and the results are consistent with the reported clinical data [13]. Moreover, if an additional type of the device fixation due to adhesion between the maxillofacial surgery plate and a bone is imposed (if, for example, later post operational stages are considered), the fatigue life predictions might considerably vary, and thus, yielding insufficient fatigue strength of the tested appliance. Therefore, the application of standard testing procedures for the medical devices should be treated with care, since realistic deviations in loads and boundary conditions may have a dramatic influence on the device reliability.

For the future progress in the field, it is essential to estimate the possible effect of crystallographic texture inherited form the deformation process of nanostructuring. This issue can be critical for the components fabricated of the materials with lower crystal symmetry (such as Ti having the HCP lattice). Note that multi-scale computational approaches [23,35,36] developed to describe nanostructuring processes of Ti materials by ECAP demonstrated that macroscale simulation coupled with polycrystal models provided better description of its deformation behavior. They also allowed to take into consideration the texture effects important to account for the anisotropic properties [37]. Thus, further systematic simulation and validation studies are required to clarify these above issues as well to develop reliable procedures for the combined virtual testing of advanced medical products.

## 5. Conclusions

We propose pathways to FEM-driven design of advanced miniaturized medical implants fabricated of biocompatible materials with considerably enhanced mechanical performance. We present numerical results for two case studies—virtual testing of a dental implant and a maxillofacial surgery plate produced of the nanostructured commercially pure Ti Grade 4. We show that the diameter of dental implants can be safely reduced down to 2.0 mm if the nano-Ti is used and the miniaturized device keeps excellent static and cyclic strength. We also demonstrate that significantly thinner devices for maxillofacial surgery can be reliably produced of the nano-Ti—the cross-section area of a standard bone-shaped maxillofacial surgery plate can be reduced by 20% without a risk of the premature fatigue failure. It is important to note that variations in loading application and fixation methods may affect the virtual fatigue tests. To sum up, the developed approach appears to be a promising tool for the simulation-aided design of new medical articles and the progress in the area requires further intensive research involving multi-scale simulation approaches combined with the experimental validation.

## Figures and Tables

**Table 1:** Mechanical parameters of CG and UFG Ti Grade 4.

	CG Ti	UFG Ti
Young’s modulus, E, GPa	110	110
Poisson’s ratio	0.32	0.32
Ultimate tensile stress, MPa	730 ± 7	1255 ± 10
Yield stress, MPa	500 ± 15	1200 ± 30

**Table 2:** Results of virtual fatigue tests.

Model	Material	Force Application Angle (°)	Number of Cycles	Test Passed
2.4 mm (base model)	CG Ti	30	2,075,000	yes
		28	8,255,600	yes
		32	254,430	no
2 mm	UFG Ti	30	10^7^	yes
		28	10^7^	yes
		32	10^7^	yes
1.9 mm	UFG Ti	30	8,832,500	yes
		28	10^7^	yes
		32	80,756	no
1.8 mm	UFG Ti	30	0	no
		28	45,248	no
		32	0	no

**Table 3:** Results of virtual static strength test.

Model	Material	Max First Principal Stress (MPa)	Test Passed
2.4 mm (base model)	CG Ti	530	yes
2 mm	UFG Ti	815	yes
1.9 mm	UFG Ti	928	yes
1.8 mm	UFG Ti	1106	yes

**Table 4:** Results of the virtual fatigue tests for the maxillofacial surgery plate.

Model	Material	Load Type	Fixation Type	Number of Cycles	Test Passed
Base model	CG Ti	A	A	10^6^	Yes
		A	B	1.4 × 10^6^	Yes
		B	A	10^6^	Yes
		B	B	0	No
Cross-section reduced by 20% (UFG20)	UFG Ti	A	A	10^7^	Yes
		A	B	10^7^	Yes
		B	A	10^7^	Yes
		B	B	0	No
Cross-section reduced by 30% (UFG30)	UFG Ti	A	A	10^7^	Yes
		A	B	8.05 × 10^6^	Yes
		B	A	90,295	No
		B	B	0	No
Cross-section reduced by 40% (UFG40)	UFG Ti	A	A	10^7^	Yes
		A	B	0	No
		B	A	0	No
		B	B	0	No

## Data Availability

The raw and processed data required to reproduce these results are available by a reasonable request.
